# Therapeutic adherence and competence scales for Developmentally Adapted Cognitive Processing Therapy for adolescents with PTSD

**DOI:** 10.3402/ejpt.v6.26632

**Published:** 2015-03-18

**Authors:** Jana Gutermann, Franziska Schreiber, Simone Matulis, Ulrich Stangier, Rita Rosner, Regina Steil

**Affiliations:** 1 Department of Clinical Psychology and Psychotherapy, Goethe University, Frankfurt am Main, Germany; 2 Department of Clinical and Biological Psychology, Catholic University of Eichstätt-Ingolstadt, Eichstätt, Germany

**Keywords:** Cognitive Processing Therapy, D-CPT, PTSD, therapeutic adherence, therapeutic competence, treatment integrity, adolescents

## Abstract

**Background:**

The assessment of therapeutic adherence and competence is often neglected in psychotherapy research, particularly in children and adolescents; however, both variables are crucial for the interpretation of treatment effects.

**Objective:**

Our aim was to develop, adapt, and pilot two scales to assess therapeutic adherence and competence in a recent innovative program, Developmentally Adapted Cognitive Processing Therapy (D-CPT), for adolescents suffering from posttraumatic stress disorder (PTSD) after childhood abuse.

**Method:**

Two independent raters assessed 30 randomly selected sessions involving 12 D-CPT patients (age 13–20 years, *M* age=16.75, 91.67% female) treated by 11 therapists within the pilot phase of a multicenter study.

**Results:**

Three experts confirmed the relevance and appropriateness of each item. All items and total scores for adherence (intraclass correlation coefficients [ICC]=0.76–1.00) and competence (ICC=0.78–0.98) yielded good to excellent inter-rater reliability. Cronbach's alpha was 0.59 for the adherence scale and 0.96 for the competence scale.

**Conclusions:**

The scales reliably assess adherence and competence in D-CPT for adolescent PTSD patients. The ratings can be helpful in the interpretation of treatment effects, the assessment of mediator variables, and the identification and training of therapeutic skills that are central to achieving good treatment outcomes. Both adherence and competence will be assessed as possible predictor variables for treatment success in future D-CPT trials.

To interpret accurately the results of treatment outcome studies, treatment integrity must be ascertained because it ensures internal validity (Waltz, Addis, Koerner, & Jacobson, 1993). Furthermore, aspects of treatment integrity can be analyzed as potential mediators of treatment outcome. These can help elucidate the mechanisms of treatment success and help identify and teach central therapeutic skills (Perepletchikova & Kazdin, 2005). Treatment integrity comprises treatment adherence, competence, and differentiation. Adherence refers to “the extent to which a therapist use[s] interventions and approaches prescribed by the treatment manual and avoid[s] the use of intervention procedures proscribed by the manual” (Waltz et al., 1993) and therefore serves as a “manipulation check” in treatment outcome studies. Competence refers to “the level of skill shown by the therapist in delivering the treatment” (Waltz et al., 1993). A competent therapist not only administers the treatment as prescribed in the manual but also considers matters that are relevant to the therapeutic context, such as client variables (e.g., age, symptom severity) and/or the stage of therapy. Treatment differentiation requires that different treatments are distinguishable from one another with regard to critical aspects.

Despite the importance of treatment integrity, it is often neglected in psychotherapy research (Perepletchikova, Treat, & Kazdin, 2007), particularly in studies of children and adolescents (Webb, Auerbach, & DeRubeis, 2012) and in posttraumatic stress disorder (PTSD) outcome research (Barber, Triffleman, & Marmar, 2007). In addition to the fundamental elements of treatment integrity, such as adequate therapist training/supervision and the provision of a treatment manual, ratings of videotaped sessions are recommended to assess treatment adherence and therapeutic competence (Bellg et al., 2004). However, such ratings are difficult, time-consuming, and expensive (Perepletchikova, Hilt, Chereji, & Kazdin, 2009). Furthermore, when adherence and/or competence are assessed using adherence and competence scales, the psychometric properties of those measures are frequently not reported (Perepletchikova et al., 2007).

Only a few trials have analyzed treatment integrity in child and adolescent psychotherapy samples. A recent review of the assessment of treatment integrity variables in studies on youth with externalizing behavioral problems concluded that such analyses are rare, are mostly limited to treatment adherence, and lack information on the psychometric properties of the instruments (Goense, Boendermaker, Van Yperen, Stams, & Van Laar, 2014). A newly developed rating scale for the assessment of therapeutic competence in child and adolescent samples was recently published. This instrument, the Cognitive Behaviour Therapy Scale for Children and Young People (CBTS-CYP) (Stallard, Myles, & Branson, 2014), measures general competencies in CBT but not specific competencies for distinct treatment manuals.

Relationships between therapeutic adherence/competence and treatment outcomes are of increasing interest in adult psychotherapy research. The extant literature suggests that treatment integrity is related to treatment outcome (Webb, DeRubeis, & Barber, 2010). The assessment of both treatment and/or disorder-specific competencies and global therapeutic competencies is recommended (Barber, Sharpless, Klostermann, & McCarthy, 2007). Global competencies indicate the general ability of the therapist to adequately support the patient, such as by structuring the therapy properly or by high interpersonal competences, apart from a specific treatment or disease. However, recent research indicates that treatment- and disorder-specific competencies, in particular, are more likely to predict treatment response than global competencies (Ginzburg et al., 2012). In a study on social phobia, Ginzburg et al. (2012) showed that interventions designated to directly address symptoms of social phobia predicted the reduction of social phobia symptoms at the end of therapy particularly well.

Regarding treatment adherence, some studies use scales in the form of a checklist for the content of each session, and these checklists are frequently combined with a competence measure (e.g., did the therapist deliver the designated content of the session and, if so, how competently did he do so?) (Barber, Liese, & Abrams, 2003). Other studies use a global adherence measure developed to assess adherence at every possible treatment session that includes a separate measure for therapeutic competence (von Consbruch, Clark, & Stangier, 2012). These global measures allow for the random selection of videos from a treatment course; rating randomly selected videos is less time-consuming and easier to implement in both research and training settings compared with rating every therapy session (Barber, Triffleman, et al., 2007). Separate assessment of adherence and competence helps differentiate between the two aspects, which seems to be a challenge for raters because a number of studies have demonstrated high correlations between adherence and competence (e.g., Ginzburg et al., 2012, *r*=0.69; Shaw et al., 1999, *r*=0.63–0.66). It is likely that adherence and competence are related but also that raters frequently have problems differentiating between the two (Barber, Triffleman, et al., 2007). Whereas correlations between adherence and competence are natural, it is important for assessments of treatment integrity to distinguish between these aspects (Barber, Triffleman, et al., 2007).

The approach used to assess adherence and competence is closely connected to the treatment that is being evaluated (Barber, Triffleman, et al., 2007). In treatments that aim for a flexible, modular, hierarchical intervention process tailored to the individual patient (in contrast to a fixed sequence of interventions for all patients), adherence measures that use a checklist for each session are not adequate because it cannot be known which intervention will be applied at each session. Furthermore, the therapist's competence is closely connected to the flexible adaptation of treatment to the patient's problems; such treatment approaches include both compulsory and optional elements. In particular, assessing therapeutic competencies regarding treatment for severe PTSD or borderline personality disorder is challenging because those treatments must have flexible responses to crises, such as dialectical behavior therapy (Bohus et al., 2013; Matulis, Resick, Rosner, & Steil, 2014).

The relationship between adherence/competence and outcome has been analyzed in only a few trials on the treatment of adolescents. Previous analyses have been limited to adherence to the treatment manual, which is associated with greater treatment effects pertaining to drug use, externalizing behavior, and family functioning in drug-abusing adolescents (Hogue et al., 2008; Robbins et al., 2011). Intermediate levels of adherence predicted declines in internalizing behaviors, whereas therapeutic competence did not predict outcomes (Hogue et al., 2008). Huey and colleagues analyzed the impact of treatment adherence for juvenile offenders and demonstrated larger effects following adherent treatment (Huey, Henggeler, Brondino, & Pickrel, 2000). In youths with anxiety disorders, treatment integrity showed small but significant correlations with improvements in psychopathology (Podell et al., 2013). The measures used in these studies closely correspond to the specific treatment manuals and are therefore not directly transferable to other treatments and disorders.

To the best of our knowledge, there are no studies of the relationship between treatment adherence and/or competence and treatment outcomes for youths with PTSD. We found only one study of an adult PTSD sample treated with a Gestalt-derived treatment that analyzed treatment integrity and its role as a predictor of treatment outcome (Paivio, Holowaty, & Hall, 2004). It showed that adherence—and not competence—predicted treatment success. Furthermore, only 54% of the sample met the DSM-IV PTSD criteria, and adherence and competence were rated by non-expert judges. For adolescents with PTSD, only one study systematically assessed adherence (Gilboa-Schechtman et al., 2010), and another examined both adherence and competence (Ford, Steinberg, Hawke, Levine, & Zhang, 2012). In the latter study, most of the patients had experienced abuse but did not disclose childhood sexual and/or physical abuse (CSA/CPA).

None of the existing studies on the treatment of adolescents with PTSD solely after CSA/CPA (e.g., Danielson et al., 2010, 2012; Foa, McLean, Capaldi, & Rosenfield, 2013; Matulis et al., 2014) have analyzed the impact of adherence and/or competence on outcomes. To the best of our knowledge, Foa et al. (2013) and Danielson et al. (2010, 2012) are the only studies on adolescents and Cohen, Mannarino, Perel, and Staron (2007) is the only study on children and adolescents with PTSD after CSA or CPA to assess treatment adherence in the sense that they reviewed actual therapy sessions.

To date, the impact of adherence and competence on treatment success among adolescents is unknown. Recent recommendations propose that therapeutic adherence and competence should be ascertained through direct ratings of videotapes using a valid and reliable measure that corresponds closely to the given treatment manual (Barber, Triffleman, et al., 2007). Adequate measures of competence can be found in the literature, such as the Cognitive Therapy Scale (CTS) (Weck, Hautzinger, Heidenreich, & Stangier, 2010), which is derived from the original CTS (Young & Beck, 1980) and its revision (Blackburn et al., 2001). The CTS and its adaptions are widely used to assess therapeutic competence in various settings (Simons et al., 2010; Strunk, Brotman, DeRubeis, & Hollon, 2010). However, no adaption exists to measure the particular competencies required to treat adolescents or patients with PTSD.

We developed the Therapeutic Adherence Scale (TAS) and the Therapeutic Competence Scale (TCS) for use with Developmentally Adapted Cognitive Processing Therapy (D-CPT) for adolescents with PTSD following CSA/CPA. D-CPT is based on a combination of Cognitive Processing Therapy (CPT) (Resick & Schnicke, 1992, 1993), a PTSD treatment that has been extensively researched in adults, and training in emotion regulation and other aspects. CPT has proven to be effective and efficient in various settings following different types of trauma (Watts et al., 2013). D-CPT showed good results in a pilot study (Matulis et al., 2014). The newly developed scales measuring therapeutic competence and adherence in D-CPT were tested for relevant psychometric properties.

## Methods

### Developmentally Adapted Cognitive Processing Therapy

D-CPT comprises 30–36 sessions delivered in an intensive outpatient setting for 5–6 months. It is structured into four phases (for a detailed description, see Matulis et al., 2014 as well as additional information in the Supplementary file).

### The TAS for D-CPT

The TAS was derived from the treatment manual for D-CPT (Matulis et al., 2014) and takes into account the CPT Therapist Adherence and Competence Protocol—Revised (Resick, 2012) and the Cognitive Therapy Adherence Scale for Social Phobia (CTAS-SP) (von Consbruch et al., 2012). A three-point Likert scale was used (0=not adherent, 1=adherent to some extent, 2=adherent) with descriptions of both complete non-adherence and complete adherence. All items were created to be applicable to each treatment session. Ten items reflected the therapist's adherence. One item reflected whether interventions from other therapy orientations were used (Barber, Triffleman, et al., 2007). The last item assessed global adherence in the therapy session using a seven-point Likert scale ranging from 0 to 6 (0=not adherent to 6=very adherent).

### The TCS for D-CPT

The TCS consists of an adaption of previous versions of the CTS (Weck et al., 2010) plus seven newly developed items specific to D-CPT. The CTS used by Weck et al. (2010) showed good psychometric properties, with intraclass correlation coefficients (ICCs) between 0.66 (for resource activation) and 0.95 (for agenda), including an ICC of 0.73 for overall session competence. The CTS items were adapted for adolescent PTSD treatment. For example, item 2, which examines “dealing with questions, problems,” includes a statement that in adolescent PTSD patients such problems can manifest as dissociation, increased arousal, or mind wandering. The items specific to D-CPT address issues of patient autonomy, facilitating cooperation (Creed & Kendall, 2005; Matulis et al., 2014; Podell et al., 2013), handling severe stress and intense emotions, balancing validation strategies with change-oriented interventions, and using contingency management. These items were added in keeping with recommendations to distinguish global from specific competences (Barber, Sharpless, et al., 2007). The last TCS item assesses global competence in the session. All items are rated on a seven-point Likert scale (0=poor competence to 6=excellent competence). Every second point on the Likert scale is illustrated with text; in-between points are adequate if neither the higher nor the lower description is completely adequate. All items were constructed to be applicable to each treatment session.

To estimate the effect of sympathy toward the therapist on competence ratings, the raters were asked to assess how much they liked the therapist after watching the video for 10 min and to actively attempt to exclude this opinion from their ratings. Furthermore, the raters were asked to evaluate the patient's commitment to the treatment and the difficulty of treating the patient. All the above-mentioned aspects should be considered when rating therapeutic competence (Waltz et al., 1993).

### Scale development and rater training

It was decided *a priori* to construct two separate scales and to develop items within the scales that permitted reviewing videos for randomly chosen (and not predetermined) sessions. Despite the phase-based nature of D-CPT, we constructed adherence and competence items that should be relevant to any treatment session. In the first step, two raters (first and second author) rated 10 randomly selected videotapes from the pilot trial of a larger randomized controlled trial (RCT) of D-CPT. These videos were excluded from subsequent analyses. Problems with the use of the scales were noted and discussed, and some items were modified. The two raters had 2.5 and 3.5 years of clinical experience. Both had treated patients with D-CPT under supervision, and both were intensively trained (46 h) in D-CPT by its developers.

To prevent the raters from drifting during the evaluation process, the ratings of every fifth video were compared and the differences were discussed, which is consistent with previous research studies (von Consbruch et al., 2012; Weck et al., 2010). However, the ratings were not changed after this comparison. Therefore, the ratings were assessed independently.

### Content validation

For content validation, three experts in D-CPT (the third, fifth, and last author) were asked to provide feedback on the scales. They evaluated the relevance and appropriateness of each item on a scale from 0 (not at all relevant/appropriate) to 3 (extremely relevant/appropriate). No items were missing, and additions to and suggestions for the item descriptions were considered.

### Framework of analysis of psychometric properties of the scales

The two scales were examined during the pilot phase of a multicenter RCT to evaluate the effectiveness of D-CPT compared with treatment as usual (Rosner, Konig, Neuner, Schmidt, & Steil, 2014) in adolescents who had experienced CSA/CPA (for inclusion and exclusion criteria see the Supplementary file). The local ethics committee approved the pilot trial. Both assessment and treatment were offered in three university outpatient clinics in Germany. Patients were mainly referred by hospitals, private psychologists, social workers, and caregivers.

### Participants and therapists

Videotapes from 12 patients and 11 therapists were included in the ratings, and 30 sessions were randomly selected. To ascertain the external validity of the scales for different patients and different therapists, up to three videos per patient and up to four videos per therapist were rated.

The patients were 91.76% female with a mean age of 16.75 years (*SD=*2.42). All participants were Caucasian. PTSD diagnosis was related to CPA in three cases, and to CSA in nine cases. Five patients had previously had outpatient psychological treatment, and five had previously had inpatient psychiatric treatment.

The main treatment outcome was the reduction of PTSD symptoms as assessed with the clinician-administered PTSD Scale (CAPS-CA; Nader et al., 1996). Two of the twelve patients had to be excluded from the pilot trial in retrospect because of a lacking validity of the reported trauma.

All therapists were psychologists (four at the masters level, and seven on the doctoral level) with a mean of 61.09 months of clinical experience (*SD*=24.18), and 81.82% were female. Nine were licensed cognitive behavioral therapists, and two were still in training. All the therapists were trained and supervised in D-CPT by the method's developers.

### Data analyses

All item scores represent the averages of the two rating values from both raters. Means and standard deviations are presented for each item as well as for the experts’ ratings regarding relevance and appropriateness. To analyze inter-rater reliability, ICCs were computed for all items using Model 2 [ICC_(2,2)_], following the recommendations from Shrout and Fleiss (1979). The 95% confidence interval was used to determine statistical significance. The ICCs were calculated based on all the videotapes (*N*=30) rated by both raters. Absolute consistency between the two raters was required. ICCs exceeding 0.75 were considered good (Portney & Watkins, 2008).

The internal consistency of the scales was tested by calculating Cronbach's alpha coefficient. Scores exceeding 0.7 are acceptable (Cortina, 1993). The relationship between adherence and competence (total scores of each rating), in addition to the relationship between the reduction of PTSD symptoms as indicated by CAPS-CA (Nader et al., 1996)—pre-treatment versus post-treatment—and the mean adherence/mean competence were assessed by calculating Pearson's correlation coefficient. All analyses were conducted using IBM SPSS^®^, Version 22.

## Results

Table 1 shows the frequency with which each of the 30 D-CPT session was rated. Table 2 shows the frequency with which each of the 44 different interventions of D-CPT was identified by the rater (Item 4 of the TAS). The rate of agreement between the two raters regarding the occurrence of interventions was 99.20%.

**Table 1:** Frequency with which each session had been rated within the 30 ratings

Phase	Session	Rating of frequency
Commitment phase		
	1	1
	2	2
	3	2
	4	2
	5	1
Emotion regulation phase		
	6	0
	7	2
	8	1
	9	2
	10	0
	11	1
CPT phase		
	12	5
	13	0
	14	0
	15	1
	16	1
	17	0
	18	0
	19	1
	20	2
	21	0
	22	0
	23	0
	24	2
	25	1
	26	0
Development-assignment phase and treatment termination		
	27	0
	28	0
	29	1

**Table 2:** Frequency with which each intervention had been identified in the 30 ratings by the first author (Item 4 of the Treatment Adherence Scale [TAS])

Phase of therapy	Frequency
*Commitment phase*	
Fostering commitment and the therapeutic relationship through empathy, warmth, emphasis of the freedom of choice of the patient, etc.	1
Therapy contract	2
Emergency plan	2
Introduction of the diary card	3
Lifeline	4
Formulation of goals for therapy	3
Establishing contacts with parents/care-takers and relevant institutions	1
Preparation and planning for intense periods	1
*Emotion regulation phase*	
Psychoeducation concerning severe stress/dissociation/skills/emotions	4
Triggers, early warning signals and recognition of stress and severe stress, and, if necessary, dissociation	3
Stress protocol, stress curve	3
Behavioral analysis	1
Functional and dysfunctional (e.g., self-harm, addiction, suicidality) handling of previously existing severe stress	1
Pros and cons, advantages and disadvantages of ways in which previously existing severe stress had been addressed	2
Selection of strategies that are to be reduced, development of alternatives for dysfunctional behavior	3
In case of less dysfunctional behavior, development of preventive strategies	1
Conveying techniques for stress regulation and tolerance	4
Definition of skills, expansion and strengthening of useful skills	4
Encouragement of autonomous implementation by the patient	1
Labeling of and dealing with feelings, “star of feelings,” network of feelings	2
Preparation and planning for intense periods	0
Emergency suitcase and arrangements	1
*CPT phase*	
Psychoeducation about PTSD, trauma memory, relationship between thoughts and feelings	5
Addressing the patient's concerns regarding the handling of trauma	3
Working on the Impact Statement	0
Reading and working on the TraumaReport	2
Collecting Stuck Points, identifying essential Stuck Points, questioning, working on (Assimilation and Over-Accommodation), possible elaboration of the function	6
Relationship between thoughts and feelings, ABC-Schema	4
Implementing and evaluating useful questions	5
Checking convictions	5
Guided discovery	5
Socratic Dialogue	3
Graphic illustrations, e.g., guilt-pie	0
4-area-schema	1
Looking for alternative explanations for the evaluation	2
Devil's Advocate	0
Implementing and evaluating thought patterns	2
Dealing with specific modules: safety, trust, control, being worthy, and closeness	2
If needed, facilitating the autonomous use of learned techniques in situations of severe stress	0
*Development-assignment phase and treatment termination*	
Dealing with topics that are relevant to the adolescent's development, such as choice of partner/re-victimization, choice of career/school/education, detachment of parents/developing autonomy	1
Relapse prevention, intervention to facilitate mental health (if no topic area seems relevant to the adolescent's development)	0
Network tasks, initiate continuative measures	0
Renewed Impact Statement	0
Evaluation of therapy	0

The range, mean, and standard deviation values for all items and for the experts’ ratings on relevance and appropriateness—in addition to the ICCs for both raters and total scores—on all competence and adherence items are presented in Tables 3 and 4, respectively. The assessments of the three experts regarding relevance and appropriateness of the items demonstrate that all items were considered relevant and appropriate, with ranges between 2 (very relevant/appropriate) and 3 (extreme relevant/appropriate) for each adherence and competence item, respectively.

**Table 3:** Intraclass correlation coefficient, range, mean and standard deviation of items and mean and standard deviations of expert ratings for the Therapeutic Adherence Scale (TAS) for Developmentally Adapted Cognitive Processing Therapy (D-CPT)

	Item	ICC_(2,2)_	Min/Max	*M (SD)*	Relevance *M (SD)*	Appropriateness *M (SD)*
1	Agenda	0.96***	0/2	1.42 (0.78)	2.67 (0.58)	3.00 (0.00)
2	Reviewing previously set homework	0.90***	0/2	1.22 (0.87)	2.67 (0.58)	2.67 (0.58)
3	Reviewing weekly protocol	0.96***	0/2	1.20 (0.91)	2.00 (0.00)	2.67 (0.58)
4	Implementation of intended interventions	0.76***	1/2	1.85 (0.33)	3.00 (0.00)	2.67 (0.58)
5	Phase reference	1.0	1/2	1.87 (0.35)	2.33 (0.58)	2.33 (0.58)
6	Use of treatment materials	0.84***	0/2	1.75 (0.50)	2.33 (0.58)	3.00 (0.00)
7	Cognitive approach and reference to the PTSD disorder model	0.78***	0/2	1.43 (0.61)	3.00 (0.00)	3.00 (0.00)
8	Identification and modification of avoidant behavior	0.84***	0/2	1.77 (0.55)	3.00 (0.00)	2.67 (0.58)
9	Homework setting	0.98***	0/2	1.33 (0.91)	2.00 (0.00)	3.00 (0.00)
10	Time management	0.97***	0/2	1.57 (0.72)	2.00 (0.00)	2.67 (0.58)
	Interventions from different forms of therapy	0.95***	0/2	1.88 (0.41)	2.33 (1.15)	2.00 (0.00)
	Overall session adherence	0.95***	2/6	4.30 (1.24)	2.67 (0.58)	3.00 (0.00)
	Total adherence score	0.95***	8.5/20.0	15.40 (3.17)		

*Note*: ICC_(2,2)_=Intraclass correlation coefficients for both raters; Min=lowest score on the ratings on a scale from 0 to 2; and Max=highest score on the ratings on a scale from 0 to 2. Relevance and appropriateness were assessed on a scale ranging from 0 to 3 for each item.

*** *p<*0.001.

**Table 4:** Intraclass correlation coefficient, range, mean and standard deviation of items and mean and standard deviations of expert ratings for the Therapeutic Competence Scale (TCS) for Developmentally Adapted Cognitive Processing Therapy (D-CPT)

	Item^a^	ICC_(2,2)_	Min/Max	*M (SD)*	Relevance *M (SD)*	Appropriateness *M (SD)*
1	Agenda	0.93***	0/5.5	2.63 (1.49)	2.33 (0.58)	3.00 (0.00)
2	Dealing with questions, problems, objections, and reactance	0.80***	2/5	3.95 (0.98)	3.00 (0.00)	3.00 (0.00)
3	Clarity of communication	0.81***	2/6	4.08 (0.97)	2.33 (0.58)	3.00 (0.00)
4	Pacing and efficient use of time	0.88***	1/6	3.73 (1.32)	2.67 (0.58)	2.33 (0.58)
5	Interpersonal effectiveness	0.79***	2/5.5	3.97 (1.01)	3.00 (0.00)	2.00 (1.00)
6	Resource orientation	0.91***	1/5.5	3.53 (1.18)	2.00 (0.00)	3.00 (0.00)
7	Reviewing homework	0.98***	0/5	2.42 (1.80)	2.00 (0.00)	2.67 (0.58)
8	Use of feedback and summaries	0.78***	0/4.5	2.00 (1.23)	2.67 (0.58)	3.00 (0.00)
9	Guided discovery	0.83***	1/5.5	2.82 (1.18)	2.67 (0.58)	2.67 (0.58)
10	Focus of cognitive model	0.86***	0/5.5	3.72 (1.26)	3.00 (0.00)	2.67 (0.58)
11	Rationale/transparency	0.85***	1/6	3.80 (1.14)	2.67 (0.58)	2.33 (1.15)
12	Selection of appropriate strategies	0.89***	1.5/6	4.17 (1.04)	2.67 (0.58)	2.33 (0.58)
13	Implementation of techniques	0.86***	1/5.5	3.67 (1.20)	2.33 (0.58)	2.67 (0.58)
14	Homework setting	0.93***	0/6	2.70 (1.86)	2.33 (0.58)	3.00 (0.00)
	*D-CPT specific competences*					
15	Dealing with severe stress	0.86***	2/5.5	4.08 (0.96)	2.67 (0.58)	2.66 (0.58)
16	Dealing with emotions	0.87***	1.5/5.5	3.78 (1.12)	2.67 (0.58)	3.00 (0.00)
17	Use of validation strategies	0.87***	1/6	3.80 (1.32)	2.33 (0.58)	2.67 (0.58)
18	Use of change-oriented interventions	0.88***	1/5	3.50 (1.17)	2.67 (0.58)	2.33 (0.58)
19	Consideration of autonomy	0.76***	1/5	3.57 (1.06)	2.67 (0.58)	2.33 (0.58)
20	Facilitating cooperation	0.90***	0.5/5.5	3.30 (1.35)	3.00 (0.00)	3.00 (0.00)
21	Contingency management	0.79***	1/5	3.53 (1.16)	2.67 (0.58)	3.00 (0.00)
	Overall session competence	0.94***	1/5	3.70 (1.17)	3.00 (0.00)	3.00 (0.00)
	Patient commitment	0.77***	2/5	3.38 (0.90)	2.33 (0.48)	2.67 (0.48)
	Patient difficulty	0.72**	1.5/4.5	3.08 (0.79)	2.33 (0.58)	2.33 (0.58)
	Total competence score	0.94***	29/103	72.73 (19.29)		

*Note*: ICC_(2,2)_=Intraclass correlation coefficient for both raters; Min=lowest score on the ratings on a scale from 0 to 6; and Max=highest score on the ratings on a scale from 0 to 6. Relevance and appropriateness were assessed on a scale ranging from 0 to 3 for each item.

a The first 14 items consist of the Cognitive Therapy Scale (Weck et al., 2010).

** *p*<0.01

*** *p<*0.001.

Regarding adherence, on a scale ranging from 0 to 2, all medium values were above 1. Regarding competence, some item means were considerably lower than medium (e.g., *use of feedback and summary* and *reviewing previously set homework*), whereas most items, particularly treatment-specific competence items, were above medium. When analyzing standard deviations, it is notable that categories between 3 and 5 on the seven-point Likert scale for competence were mostly used by the raters.

Cronbach's alpha coefficient for the TAS was 0.59. Three items yielded low coefficient values and contributed to a lower total score: item 1 (Agenda), item 8 (Identification and Modification of Avoidance Behavior), and item 10 (Time Management). Cronbach's alpha coefficient for the TCS was 0.96. No items were removed from the scales. Cronbach's alpha coefficient was also separately calculated for the seven items specific to D-CPT (Item 15–21) and measured 0.95. The adherence and competence sum scores were highly correlated (*r*=0.65, *p*<0.001). The mean adherence and mean competence ratings combined for all ratings of each patient from both raters were highly, yet non-significantly, associated with reduction in PTSD symptoms (*r*=0.61, *p*=0.059 and *r*=0.45, *p*=0.189, respectively).

## Discussion

This study is the first to develop and pilot an adherence and competence scale for the treatment of adolescents in an innovative program (D-CPT) recently developed for PTSD after CSA/CPA. The results indicate that both scales are relevant, adequate, and useful for reliably assessing therapeutic adherence and competence. The items of both scales were considered relevant and appropriate by experts, with the disorder-specific competence items showing particularly high relevance and appropriateness. Furthermore, the concordance between raters in the application of the scale was excellent for the entire scale and for single items, indicating reliable assessment of the specific aspects of competence and adherence for D-CPT. Both will be assessed as possible predictor variables of treatment success in future trials on D-CPT. The good reliability of single items on the scale allows for examining and defining certain aspects in the treatment of adolescent PTSD patients that might have a specific impact on the treatment effect, as shown in previous studies (Ginzburg et al., 2012). The ICCs for items adapted from the CTS (items 1–14 of the TCS) were comparable to (Weck et al., 2010) or higher than (von Consbruch et al., 2012) those reported in previous studies. The seven items specific to D-CPT also showed excellent inter-rater reliability and internal consistency in the subgroup analysis. With regard to content, the total range of categories (0–6 in the competence scale and 0–2 in the adherence scale) was not always utilized by the raters. This might be caused not only by the small sample size with restricted variance but also by the multiple efforts in the pilot phase of the large RCT study to ensure treatment adherence and competent delivery of the treatment. We assume that as the study progresses, the categories will be used more widely.

Furthermore, the TCS showed excellent internal consistency, which might also indicate that some items on this scale are redundant or measure highly interrelated competencies. This redundancy is a common problem in treatment integrity research (Blackburn et al., 2001; Stallard et al., 2014). By contrast, the internal consistency of the TAS was less positive, particularly for items 1 (Agenda), 8 (Identification and Modification of Avoidance Behavior), and 10 (Time Management). These items contributed to the low consistency scores, which may be due to the restricted variance in the small sample of this study, and these items highlight components that should continually be focused. However, considering the brevity of the scale and that Cronbach's alpha coefficient always depends on the length of the scale (Cortina, 1993), it is in an acceptable range. In future trials, the assessment of the interventions provided in the treatment manual (particularly item 4) should be undertaken in greater detail using distinct items within the TAS, assessing key components during different phases of the treatment, which might lead to higher internal consistency. However, it also must be considered that Cronbach's alpha might not apply to an adherence measure because it is measuring related but independent actions by the therapist rather than a single unidimensional construct.

As avoidance is a core symptom of PTSD, and the identification and modification of often subtle signs of avoidance are complex and important tasks, the competence of the therapist in identifying and modifying avoidance behavior was also assessed within the TCS, next to the TAS.

Consistent with previous research (Barber, Triffleman, et al., 2007), the interrelatedness of adherence and competence was high, indicating that these constructs are closely connected but not identical; the shared variance was only 42%. The high but non-significant correlations shown between adherence/competence and treatment outcome must be regarded as preliminary. Nonetheless, they replicate previous findings regarding these relationships (Webb et al., 2010).

The generalizability of our findings is restricted by several limitations. First, in the current study, two to four treatment sessions per therapist were rated. We cannot exclude the possibility that repeated ratings of the same therapist may have artificially increased inter-rater conformity. Thus, future studies should apply these scales to larger samples of both patients and therapists. Second, the therapists in this study worked in different centers; however, the sample was too small to control for possible center effects. The latter will be addressed in the large RCT. Because of the limited number of videos analyzed, important further validity analyses, such as concurrent, criterion, and predictive validity, were not possible and must wait for the larger sample and set of analyses.

One limitation is directly connected with the design of the TAS. We assessed adherence to the treatment manual in one multi-item rating (item 4). Unfortunately, within this item, we have not collected data on interventions that were omitted within our pilot ratings. We will assess this information within the ratings of the large RCT that is currently being conducted. Furthermore, limitations regarding the independence between raters and ratings must be considered. The inter-rater reliability was demonstrated with only two raters who were involved in the treatment study because each had treated one pilot patient. Previous studies have strongly recommended that expert raters should be used, particularly in the assessment of therapeutic competence (Weck, Weigel, Richtberg, & Stangier, 2011). Therefore, we decided to use clinically experienced and trained raters and accepted their relationship with the study. Their objectivity was fostered by items on the scales (sympathy, patient difficulty, and motivation) and by exclusion from group supervision. In future analyses, the assessment of patient difficulty and motivation should be used to statistically control for those effects. To the best of our knowledge, no study has yet evaluated the relationship between sympathy for the therapist and treatment integrity ratings. It must be questioned whether raters are able to follow the instruction to avoid being influenced by their sympathy for the therapist. The sympathy rating might be influenced by the therapist′s competence, or vice versa. These issues threaten the validity of integrity ratings and should be addressed in future research.

Content validation in this study was obtained from clinical experts involved in the treatment rather than from independent experts. This limitation occurred because D-CPT is a new treatment and only these clinical experts had full insight into its structure. Nevertheless, obtaining opinions from independent experts in PTSD and/or adolescent psychotherapy who are not involved in the treatment study would be worthwhile.

The homogeneity of our sample with regard to sex and ethnicity is another limitation. Most of our patients (nine of twelve patients) had experienced CSA, with girls reporting higher rates of CSA than boys (Pereda, Guilera, Forns, & Gomez-Benito, 2009). Homogeneity with regard to ethnicity corresponds to Germany's demographics in this regard, where only a minority of youth is non-Caucasian.

The psychometric properties of the presented scales are good, whereas in other studies, these properties are often poor or not reported. Compared with the newly developed CBTS-CYP for CBT in children and young people (Stallard et al., 2014), our scales focus more directly on adolescents and are specific for D-CPT.

In future studies, identifying the predictive value of both scales for treatment success will be crucial. Research demonstrates that rather large samples are necessary to identify the potentially small to moderate relationships between adherence/competence and treatment outcomes (Webb et al., 2010). Therefore, these scales will be used to analyze this relationship in a multicenter RCT that is currently being conducted on 90 adolescent PTSD patients. Further tests on the validity of the scales will be conducted in this larger sample, including factor analyses and correlations with external assessments. Internal consistency will be recalculated in the larger sample, particularly because of the rather low internal consistency of the TAS.

The good results on inter-rater reliability in this pilot trial indicate that the scales can be used reliably with careful training and supervision. Nonetheless, this should be confirmed by independent ratings of a sample of therapy tapes in each subsequent study. Similarly, although using an expert rater is recommended, the assessment of adherence, in particular, can be conducted by well-trained clinically inexperienced psychologists, which allows for usage in real-word clinical settings.

Notwithstanding these limitations, our preliminary findings indicate that these scales are appropriate instruments for measuring treatment adherence and competence in D-CPT. The scales may contribute to future psychotherapy research by assuring internal validity and contribute to research on adherence and competence as possible moderators of treatment success. The scales can furthermore be used for training and clinical purposes: assessing and providing feedback about therapeutic competence and adherence enables therapists and supervisors to check and improve the skills used in delivering essential elements of treatments.

## Supplementary Material

Therapeutic adherence and competence scales for Developmentally Adapted Cognitive Processing Therapy for adolescents with PTSD

Therapeutic adherence and competence scales for Developmentally Adapted Cognitive Processing Therapy for adolescents with PTSD

Therapeutic adherence and competence scales for Developmentally Adapted Cognitive Processing Therapy for adolescents with PTSD

Therapeutic adherence and competence scales for Developmentally Adapted Cognitive Processing Therapy for adolescents with PTSD

Therapeutic adherence and competence scales for Developmentally Adapted Cognitive Processing Therapy for adolescents with PTSD
