# Supplementary material for: Therapeutic adherence and competence scales for Developmentally Adapted Cognitive Processing Therapy for adolescents with PTSD
Source: Eur J Psychotraumatol. 2015 Mar 18;6:10.3402/ejpt.v6.26632. doi: 10.3402/ejpt.v6.26632 (PMC4366479; doi:10.3402/ejpt.v6.26632)
Supplement: Therapeutic adherence and competence scales for Developmentally Adapted Cognitive Processing Therapy for adolescents with PTSD [file EJPT-6-26632-s001.pdf]

Skalen zur Erfassung therapeutischer Adhärenz und Kompetenz in der Entwicklungsangepassten traumafokussierten Kognitiven Verhaltenstherapie (E-KVT) für Jugendliche mit PTSD

Hintergrund

Die Erfassung therapeutischer Adhärenz (Manualtreue) und Kompetenz wird in Psychotherapiestudien häufig vernachlässigt, insbesondere im Bereich der Kinder- und Jugendlichenpsychotherapie sowie im Bereich der Traumatherapie. Beide Variablen sind jedoch im Sinne der Sicherung interner Validität wichtig für die Interpretation von Behandlungseffekten. Darüber hinaus kann die Erforschung des Einflusses von Adhärenz und Kompetenz helfen, therapeutische Fähigkeiten zu identifizieren, die zur Erreichung guter Behandlungsergebnisse zentral sind. Die bisherige Forschung zeigt, dass moderate, aber bedeutsame Zusammenhänge von Adhärenz bzw. Kompetenz mit dem Behandlungserfolg existieren.

Ziel

Psychotherapeutische Adhärenz und Kompetenz sollten in einer neuen Behandlung für Jugendliche und junge Erwachsene mit Posttraumatischer Belastungsstörung nach körperlicher und sexualisierter Gewalt in der Kindheit, der entwicklungsangepassten traumafokussierten kognitiven Verhaltenstherapie (E-KVT), erfasst werden. Hierzu sollen Videoanalysen aus Therapiesitzungen erfolgen und mithilfe von geeigneten Skalen die Adhärenz und Kompetenz des Therapeuten beurteilt werden. Besonderer Fokus wurde hierbei neben der Erfassung allgemeiner therapeutischer Kompetenzen auf die Erfassung von störungs-, alters- und behandlungsspezifischen Kompetenzen gelegt.

Methode

Wir entwickelten, zum Teil durch Adaptation bestehender Skalen, Adhärenz- und Kompetenzskalen für E-KVT, und untersuchten diese auf ihre psychometrische Güte. Drei Experten wurden hinzugezogen zur Beurteilung der inhaltlichen Validität der Skalen. Zwei unabhängige, geschulte Rater beurteilten 30 zufällig gezogene Therapievideos (bestehend aus bis zu drei Videos pro Patient) von 12 E-KVT Patienten (13 bis 20 Jahre alt, *M* Alter = 16.75, 91.67 % weiblich), die von 11 Therapeuten in der Pilotphase einer multizentrischen randomisiert kontrollierten Studie behandelt wurden. Die Inter-Rater-Reliabilität wurde berechnet. Zur Erfassung der internen Konsistenz der Skalen wurde Cronbach’s alpha berechnet. Weiterhin wurde der Zusammenhang von Adhärenz und Kompetenz bestimmt sowie eine erste explorative Analyse zum Zusammenhang dieser Variablen mit dem Behandlungserfolg vorgenommen, beides mithilfe von Korrelationen.

Ergebnisse

Die drei Experten bestätigten die Relevanz und Eignung der Items. Alle Items für Adhärenz (ICC = .76- 1.00) und Kompetenz (ICC = .78- .98) zeigten gute bis exzellente Inter-Rater-Reliabilitäten. Die Gesamtbeurteilungen für Adhärenz und Kompetenz zeigten hervorragende Inter-Rater-Reliabilitäten (.95 und .94). Cronbach’s alpha war .59 für die Adhärenz-Skala und .96 für die Kompetenz-Skala. Der Zusammenhang zwischen Adhärenz und Kompetenz war erwartungsgemäß hoch ($r = .65$, $p < .001$). Es zeigten sich in der aufgrund der geringen Stichprobengröße vorläufigen Analyse hohe, jedoch nicht-signifikante, Zusammenhänge zwischen Adhärenz sowie Kompetenz und der Reduktion der PTSD Symptomatik der Patienten ($r = .61$, $p = .059$ in Bezug auf Adhärenz, $r = .45$, $p = .189$, in Bezug auf Kompetenz).

Schlussfolgerung

Die Skalen sind inhaltlich geeignet und ermöglichen die reliable Beurteilung der Adhärenz und Kompetenz mit Hilfe von Videoaufzeichnungen der E-KVT-Behandlung von Jugendlichen mit PTSD. Die Sicherung und Beurteilung der therapeutischen Adhärenz und Kompetenz kann neben ihrer Notwendigkeit für die Interpretation der gefundenen Behandlungseffekte auch als

Moderator des Therapieerfolgs untersucht werden. Dies kann dazu beitragen, die für den Therapierfolg zentralen therapeutischen Skills zu identifizieren und gezielt in Ausbildung und Training von Therapeuten zu fördern. Therapeutische Adhärenz und Kompetenz werden als mögliche prädiktive Variablen für den Therapieerfolg im Rahmen einer aktuell durchgeführten multizentrischen randomisiert kontrollierten Studie erfasst und untersucht. Erste vorläufige Ergebnisse deuten einen Zusammenhang zwischen der Adhärenz sowie Kompetenz mit der Symptomreduktion der Patienten an.

Citation: European Journal of Psychotraumatology 2015, 6: 26632 - http://dx.doi.org/10.3402/ejpt.v6.26632
