# Supplementary material for: Therapeutic adherence and competence scales for Developmentally Adapted Cognitive Processing Therapy for adolescents with PTSD
Source: Eur J Psychotraumatol. 2015 Mar 18;6:10.3402/ejpt.v6.26632. doi: 10.3402/ejpt.v6.26632 (PMC4366479; doi:10.3402/ejpt.v6.26632)
Supplement: Therapeutic adherence and competence scales for Developmentally Adapted Cognitive Processing Therapy for adolescents with PTSD [file EJPT-6-26632-s002.pdf]

## Therapeutic Adherence Scale (TAS) for D-CPT
## Assessment of the accordance with the manual on D-CPT

| | |
|---|---|
| **1. Agenda**<br>At the beginning of each session (within the first 15 minutes), the therapist sets an agenda together with the patient. | |
| 0 | At the beginning of the session, the therapist did not set an agenda. |
| 1 | An agenda was partly implemented. |
| 2 | At the beginning of the session, the therapist worked with the patient to set an appropriate agenda. |

| | |
|---|---|
| **2. Review of homework**<br>The therapist reviews the homework, which was assigned in the previous session. Homework assignments should always be assigned and subsequently evaluated. Exception: At the first session, you are assigning a score of 2 for this item. | |
| 0 | The therapist did not review previous homework. |
| 1 | The therapist partly implemented a review. |
| 2 | The therapist reviewed the homework. (If the patient worked on a homework assignment either alone or together with the therapist during a session, and the assignment has been clearly classified as homework by the therapist, assign a 2; If the assignments were completed during the session, but they have not been clearly identified as homework by the therapist, then assign a 1). |

| | |
|---|---|
| **3. Evaluation of the weekly protocol**<br>The therapist evaluates the weekly protocol (or introduces it in session 1). | |
| 0 | The therapist did not evaluate the weekly protocol. |
| 1 | The therapist partly evaluated the weekly protocol. |
| 2 | The therapist evaluated the weekly protocol. |

Gutermann, J., Schreiber, S., Matulis, S., Stangier, U., Rosner, R. & Steil, R. (2015). Therapeutic adherence and competence scales for Developmentally Adapted Cognitive Processing Therapy for adolescents with PTSD. Citation: European Journal of Psychotraumatology 2015, 6: 26632 - http://dx.doi.org/10.3402/ejpt.v6.26632

**4. Implementation of intended interventions**
The therapist implements the intervention for the relevant phase according to the manual (general interventions such as assignments, weekly protocol and contingency management are excluded at this point and will be evaluated as individual items).

Depending on the phase, evaluate Item 4 a, b, c or d. If interventions from other phases are implemented, please also mark these with a cross.

a) Implementation of the intended interventions in the commitment phase.
The therapist implements the interventions designated for the commitment phase.

*Fostering commitment and the therapeutic relationship through, e.g., empathy, warmth, emphasis on the freedom of choice of the patient O*
*Therapy contract O*
*Emergency plan O*
*Introduction of the diary card O*
*Life-line O*
*Formulation of goals of the therapy O*
*Establishing contacts with parents/care-takers and relevant institutions O*
*Preparation and planning for intense periods O*

| | |
|---|---|
| 0 | The therapist did not implement the interventions designated for the commitment phase. |
| 1 | The therapist partly implemented the interventions designated for the commitment phase. |
| 2 | The therapist implemented the interventions designated for the commitment phase. |

**Therapeutic Adherence Scale (TAS) for D-CPT**
**Assessment of the accordance with the manual on D-CPT**

**b) Implementation of the intended interventions in the emotion-regulation phase**
The therapist implements the interventions designated for the emotion-regulation phase.

*Psychoeducation concerning severe stress/dissociation/skills/emotions* O
*Triggers, early warning signals and recognition of stress and severe stress, and if necessary dissociation* O
*Stress protocol, stress curve* O
*Behavioral analysis* O
*Functional and dysfunctional (e.g., self-harm, addiction, suicidality) handling of hitherto existing severe stress* O
*Pros and cons, advantages and disadvantages of ways in which hitherto existing severe stress has been dealt with* O
*Selection of strategies that are to be reduced, development of alternatives for dysfunctional behavior* O
*In case of less dysfunctional behavior: development of preventive strategies* O
*Conveying techniques for stress regulation and tolerance* O
*Definition of skills, expansion und strengthening of useful skills* O
*Encouragement of autonomous implementation by the patient* O
*Labeling of and dealing with feelings, "star of feelings", network of feelings* O
*Preparation and planning for intense periods* O
*Emergency suitcase and arrangements* O

| | |
|---|---|
| 0 | The therapist did not implement the intended interventions for the emotion-regulation phase. |
| 1 | The therapist partly implemented the intended interventions for the emotion-regulation phase. |
| 2 | The therapist implemented the intended interventions for the emotion-regulation phase. |

Gutermann, J., Schreiber, S., Matulis, S., Stangier, U., Rosner, R. & Steil, R. (2015). Therapeutic adherence and competence scales for Developmentally Adapted Cognitive Processing Therapy for adolescents with PTSD. Citation: European Journal of Psychotraumatology 2015, 6: 26632 - http://dx.doi.org/10.3402/ejpt.v6.26632

## Therapeutic Adherence Scale (TAS) for D-CPT
## Assessment of the accordance with the manual on D-CPT

| c) Implementing the intended interventions for the CPT phase | |
|---|---|
| **c) Implementing the intended interventions for the CPT phase**<br>The therapist implements the intended interventions for the CPT phase.<br><br>*Psychoeducation about PTSD, trauma memory, relationship between thoughts and feelings* O<br>*Addressing the patient's concerns regarding the handling of trauma* O<br>*Editing the Impact Statement* O<br>*Reading and editing the Trauma-Report* O<br>*Collecting Stuck Points, identifying essential Stuck Points, questioning, editing (Assimilation and Over-Accommodation), possible elaboration of the function* O<br>*Relationship between thoughts and feelings, ABC-Schema* O<br>*Implementing and evaluating useful questions* O<br>*Checking convictions* O<br>*Guided discovery* O<br>*Socrates' Dialogue* O<br>*Graphic illustrations, e.g., guilt-pie* O<br>*4-area-schema* O<br>*Looking for alternative explanations for the evaluation* O<br>*Devil's Advocate* O<br>*Implementing and evaluating thought patterns* O<br>*Dealing with specific modules: safety, trust, control, being worthy, closeness* O<br>*If needed, facilitating the autonomous use of learned techniques in situations of severe stress* O | |
| 0 | The therapist did not implement the interventions intended for the CPT phase. |
| 1 | The therapist partly implemented the interventions intended for the CPT phase. |
| 2 | The therapist implemented the interventions intended for the CPT phase. |

Gutermann, J., Schreiber, S., Matulis, S., Stangier, U., Rosner, R. & Steil, R. (2015). Therapeutic adherence and competence scales for Developmentally Adapted Cognitive Processing Therapy for adolescents with PTSD.
Citation: European Journal of Psychotraumatology 2015, 6: 26632 - http://dx.doi.org/10.3402/ejpt.v6.26632

**d) Implementing the intended interventions in the development-assignment phase and treatment termination**
The therapist implements the interventions intended for the development-assignment phase and treatment termination.

*Dealing with topics that are relevant to the adolescent's development, such as choice of partner/re-victimization, choice of career/school/education, detachment of parents/developing autonomy* O
*Relapse prophylaxis, intervention to facilitate mental health (in case no topic area seems relevant to the adolescent's development)* O
*Network tasks, initiate continuative measures* O
*Renewed Impact-Statement* O
*Evaluation of the therapy* O

| | |
|---|---|
| 0 | The therapist did not implement the interventions intended for the development-assignment phase and treatment termination. |
| 1 | The therapist partly implemented the interventions intended for the development-assignment phase and treatment termination. |
| 2 | The therapist implemented the interventions intended for the development-assignment-phase and treatment termination. |

**5. Phase reference**
**A clear reference to the phases has been established** (commitment, emotion-regulation, CPT intensive, development-assignments).

| | |
|---|---|
| 0 | A clear reference was not established. |
| 1 | A clear reference was partly established. |
| 2 | A clear reference was established. |

**6. Use of treatment materials**
The therapist uses appropriate treatment materials, meaning that the therapist makes use of the specified figures, checklists, tables, protocols or models (presented on paper or flip chart, noting down results). If necessary, the therapist hands the worksheets to the patients to take home.

| | |
|---|---|
| 0 | The therapist used no materials and discussed relationships without any treatment materials, e.g., figures, checklists, tables, protocols or models, even though the therapist mentioned the relevant key aspects. |
| 1 | The therapist partly used treatment materials. |

Gutermann, J., Schreiber, S., Matulis, S., Stangier, U., Rosner, R. & Steil, R. (2015). Therapeutic adherence and competence scales for Developmentally Adapted Cognitive Processing Therapy for adolescents with PTSD. Citation: European Journal of Psychotraumatology 2015, 6: 26632 - http://dx.doi.org/10.3402/ejpt.v6.26632

| | |
|---|---|
| 2 | The therapist used suitable treatment materials or handed it to the patient to take home. |

| **7. Cognitive approach and reference to the PTSD disorder model** The therapist refers to the fundamental cognitive model by discussing the relationship of thoughts, feelings and behaviors explicitly or implicitly or refers to it. | |
|---|---|
| 0 | The therapist made no reference to the cognitive model. |
| 1 | The therapist partly made reference to the cognitive model. |
| 2 | The therapist made reference to the cognitive model. |

| 8. **Identification and modification of avoidant behavior** The therapist identifies or addresses avoidant behavior (e.g., emotional escape), i.e., escape-strategies (dissociation, self-harm), of the patient. If there is no obvious avoidant behavior in this session, award a 2. | |
|---|---|
| 0 | The therapist did not identify or address avoidant behavior. |
| 1 | The therapist partly identified or addressed avoidant behavior. |
| 2 | The therapist identified or addressed avoidant behavior. |

| **9. Assigning of homework** The therapist assigns homework, if designated by the manual. | |
|---|---|
| 0 | The therapist did not assign homework. |
| 1 | The therapist partly assigned homework. |
| 2 | The therapist assigned homework as suggested by the manual. Or the manual did not designate any homework assignments. |

| **10. Time management** The therapist meets the designated session duration of 50 minutes. Session duration in minutes: | |
|---|---|
| 0 | The therapist significantly overran the session (by more than 10 minutes) or significantly shortened the duration (by more than 10 minutes). |
| 1 | The therapist partly implemented the designated time requirement. |
| 2 | The therapist met the designated time requirement. |

Gutermann, J., Schreiber, S., Matulis, S., Stangier, U., Rosner, R. & Steil, R. (2015). Therapeutic adherence and competence scales for Developmentally Adapted Cognitive Processing Therapy for adolescents with PTSD. Citation: European Journal of Psychotraumatology 2015, 6: 26632 - http://dx.doi.org/10.3402/ejpt.v6.26632

**Therapeutic Adherence Scale (TAS) for D-CPT**
**Assessment of the accordance with the manual on D-CPT**

## Techniques external to the manual and global manual adherence

| **11. Interventions from different forms of therapy**<br>The therapist uses interventions that do not conform to the treatment concept, e.g., in-depth psychological and psychoanalytic interventions (central focus on trauma analysis or negative life events, working with transference and counter-transference, free association, working with unconscious contents and more), systemic/family therapeutic interventions (analysis of systemic relationships and interpersonal relationships in groups, use of techniques, such as family sculptures, genograms, paradox interventions) and/or elements from other psychotherapeutic treatment manuals (e.g., interventions belonging to other disorders). | |
|---|---|
| 0 | The therapist used one or more of the above-mentioned therapeutic interventions. |
| 1 | The therapist partly implemented other therapy methods. |
| 2 | The therapist did not use any of the interventions used for other therapy methods. |

| **12. Overall session adherence**<br>**Key features:** In order for the therapy standard to meet the requirements of a study, certain criteria have to be fulfilled. The assessment does not question whether the assessed therapist is a "good therapist" or whether the interventions are good or effective, but rather whether the method and implementation meet the requirements of the manual. Please indicate your overall rating of the adherence to the manual on the following scale (0-6): |
|---|
| 0 ----------- 1 ----------- 2 ----------- 3 ---------- 4 ----------- 5 ----------- 6<br>Not adherent to the manual — Great deviation — Minor deviation — Very adherent to the manual |

Gutermann, J., Schreiber, S., Matulis, S., Stangier, U., Rosner, R. & Steil, R. (2015). Therapeutic adherence and competence scales for Developmentally Adapted Cognitive Processing Therapy for adolescents with PTSD. Citation: European Journal of Psychotraumatology 2015, 6: 26632 - http://dx.doi.org/10.3402/ejpt.v6.26632
