# Supplementary material for: Therapeutic adherence and competence scales for Developmentally Adapted Cognitive Processing Therapy for adolescents with PTSD
Source: Eur J Psychotraumatol. 2015 Mar 18;6:10.3402/ejpt.v6.26632. doi: 10.3402/ejpt.v6.26632 (PMC4366479; doi:10.3402/ejpt.v6.26632)
Supplement: Therapeutic adherence and competence scales for Developmentally Adapted Cognitive Processing Therapy for adolescents with PTSD [file EJPT-6-26632-s003.pdf]

Therapeutic Competence Scale (TCS) for D-CPT
Assessment of therapeutic competence in D-CPT

Below is the detailed description of the scale used to assess the different aspects of therapeutic competence. Enter the ratings for each dimension on a separate rating sheet on a scale from 0-6. Use the following scale:

0.........	……1…..	..…..2……	.......3.........	.......4.........	…...5.........	…......6
inadequate		moderate		good		Very good

To avoid distortion, the rater should recognize his/her own opinion about the therapist that is being assessed and exclude it from the subsequent evaluation.
For this purpose, please evaluate the likeability of the therapist after 10 minutes have passed:

0.........	…...1…..	…..2……	……3.......	……4.......	……5.......	….......6
Very dislikable	Dislikable	Rather dislikable	Indifferent	Rather likable	Likable	Very likable

**1. Agenda**<br>**Key feature:** The therapist allocates the therapy time optimally by setting reasonable and realistic topics for the session. The therapist prepares the agenda in an optimal manner together with the patient. The therapist explains tasks, changes in method and the focus of the session in such a way that the patient is granted a say.	
0	The therapist did not set an agenda.
1	*between 0 and 2*
2	The therapist set a vague or incomplete agenda and serious problems became apparent (e.g., the therapist ignored the wishes of the patient).
3	*between 2 and 4*
4	In consultation with the patient (i.e., the patient was asked or consulted for feedback), the therapist worked out an agenda that is adequate for the problems of the patient (e.g., asked the patient for any additions or whether the patient agreed to the agenda). Minor problems could be identified (e.g., too many issues for a session).
5	*between 4 and 6*
6	In cooperation with the patient, the therapist worked out an agenda adequate for the available time. Priorities were set in case of too many points.

Gutermann, J., Schreiber, S., Matulis, S., Stangier, U., Rosner, R. & Steil, R. (2015). Therapeutic adherence and competence scales for Developmentally Adapted Cognitive Processing Therapy for adolescents with PTSD. Citation: European Journal of Psychotraumatology 2015, 6: 26632 - http://dx.doi.org/10.3402/ejpt.v6.26632

**2. Dealing with questions, problems, objections, and reactance**<br>**Key features:** The therapist is able to address any problems, questions and objections of the patient regarding the therapy or the therapeutic alliance. The therapist responds in a sensitive and open manner to problems, objections, as well as reactance. Possible examples of problems in therapy include increased tension, dissociation, objections, stalling, wandering, silence, mental blackouts (especially while reading the trauma narrative), and fears of the patient related to the intense trauma treatment. Depending on the patient's impairment, assign a 4 or a 5 if you cannot identify any difficulties (because it can be assumed that the therapist has prevented the occurrence of difficulties from the beginning through his/her behavior (e.g., by using the right pace, adequate language, age appropriateness)).	
0	The therapist did not recognize problems, refused them or did not try to answer questions.
1	*between 0 and 2*
2	The therapist was partially sympathetic about problems, questions and objections of the patient but did not address them in a clear and/or appropriate manner (e.g., there were remaining misunderstandings, answers were not clear, the therapist overlooked questions, the patient did not understand the answer, or the therapist got off the subject).
3	*between 2 and 4*
4	The therapist had sympathy and sensibility for the patient's problems, questions and objections and dealt appropriately with them to some extent.
5	*between 4 and 6*
6	The therapist had sympathy and high sensibility for the patient's problems, questions and objections, answered them appropriately and then checked the patient's understanding.

**3. Clarity of communication**<br>**Key features:** The therapist uses a clear style of communication. This includes clear language, adapted to the abilities of the patient, the avoidance of technical jargon and the conveyance of information in a clear, intelligible, age-appropriate manner, for example, psychoeducational information or while talking about the trauma. Notice how much the patient understands of what is being said (do not "think" too much while guessing).	
0	The therapist exaggerated the use of technical jargon and conveyed muddled information or used language inappropriate for the patient's age.
1	*between 0 and 2*
2	The therapist conveyed conclusive information, but technical language or lecturing remained. The style of questioning was confusing (long, complicated sentences, abstract expressions), or the style of communication was not appropriate for the patient.
3	*between 2 and 4*

4	For the most part, the therapist conveyed information in a clear manner.
5	*between 4 and 6*
6	The therapist showed excellent communication skills and conveyed information in a clear and well-structured manner. He facilitated the patient's understanding through the use of metaphors and stories or through the patient's personal examples.

**4. Pacing and efficient use of time**<br>**Key features:** The therapist is able to effectively distribute the session contents in relation to the agenda (time management) and establishes soft transitions between the initial, middle and final phases. The therapist adjusts the steps to the needs and cognitive abilities of the patient. The therapist avoids or terminates unproductive digressions appropriately in order to meet the time requirements. The most important intervention of the session is sufficiently prepared for and evaluated afterwards.	
0	The therapist did not try to structure the therapy time. The session seemed aimless or had an overly rigid structure. The patient completely dominated the session.
1	*between 0 and 2*
2	The pace of the procedure was reasonably well chosen, but digressions or repetitions of the therapist and/or the patient led to an inefficient use of time. Time management was unbalanced, or the session was overrun for no reason.
3	*between 2 and 4*
4	Time was used efficiently, but minor problems became apparent (e.g., unproductive discussions were ineptly interrupted). There was balanced time distribution with recognizable initial, middle and final phases.
5	*between 4 and 6*
6	Excellent time management, whereby the entire agenda was completed. The therapist used time very efficiently by tactfully limiting unimportant or unproductive discussions. The therapist chose a pace that was appropriate for the patient.

**5. Interpersonal effectiveness**<br>**Key features:** The therapist responds sensitively and appreciatively to the description of the patient's problem. Likewise, the relationship behavior of the therapist is characterized by empathy, genuineness and appreciation. The therapist is able to give the patient hope for the success of the therapy, and he creates trust, allowing the self-opening of the patient and promoting the patient's commitment to the achievement of objectives. Note: During the reading of the trauma report, restraint is usually considered as highly interpersonally effective (except during strong excitation of the patient or apparent avoidance of feelings).	
0	The therapist had poor interpersonal skills. The therapist was unable to put himself in the patient's place. The therapist intimidated, frightened or confused the patient. Thereby, the patient did not participate, lost trust and/or became hostile (e.g., the therapist appeared to be hostile, disdainful or in some other way destructive toward the patient).
1	*between 0 and 2*
2	The style of the therapist hampered his empathic understanding at times. The patient showed little trust.
3	*between 2 and 4*
4	The therapist displayed a satisfactory degree of sympathy, appreciation, and trust and gave the patent hope for change. No significant interpersonal problems were identified. The therapist was able to understand explicit and implicit massages in communication with the patient.
5	*between 4 and 6*
6	The therapist displayed excellent interpersonal effectiveness. The therapist showed an optimal degree of sympathy, appreciation, and trust and convincingly conveyed hope for change to the patient.

**6. Resource orientation**<br>**Key features:** The therapist enables the patient to perceive his positive qualities and abilities and focuses on how those can be used to achieve the set therapy outcomes.	
0	The therapist did not address the existing skills of the patient to cope with his problems. He focused on the disorder, deficits and failures, did not involve the patient in the planning and organization of the therapy and did not reinforce the patient with respect to his achieved positive changes and developments.
1	*between 0 and 2*
2	The therapist not only focused on the patient's disorder but also focused on existing skills of the patient. However, the therapist failed to note their influence on positive changes and developments in therapy.
3	*between 2 and 4*

4	With some success, the therapist activated the ability of the patient to cope with his problems. He explicitly addressed the strengths of the patient and gave him encouraging feedback about his self-initiated progress in therapy.
5	*between 4 and 6*
6	The therapist effectively activated the capability of the patient to cope with his problems. He explicitly addressed the strengths of the patient and gave feedback about the patient's self-initiated progress in therapy. The therapist gave the feedback in such a way that the patient attributed achievements/progress to his own person.

**7. Reviewing previously set homework**<br>**Key features:** The therapist allocates enough time to assess the diary card and the results of previously assigned homework and the resulting learning success. In the intensive phase, this item can also relate to whether the therapist asks the patient to read the trauma report. It also refers to the constructive discussion of problems with homework, if those were not done. Homework should be continuously assigned as well as discussed afterwards. Exception: In the first session, the only homework to fill out is the week protocol, which should be discussed in detail.	
0	The therapist did not review the homework or did not ask why the patient had not done his homework.
1	*between 0 and 2*
2	The therapist evaluated the homework, taking note of the results, but did not encourage the patient to reflect on what he learned from this experience. Although the therapist showed some competence, numerous problems could be recognized.
3	*between 2 and 4*
4	The therapist evaluated the homework in detail and was partially successful in clarifying the results or what the patient learned from the homework. Small problems are in evidence (e.g., too little time left for discussion), but in general, the therapist was competent.
5	*between 4 and 6*
6	The therapist evaluated the homework excellently, identified any difficulties, evaluated the results of the homework and worked together with the patient towards a maximum learning-benefit that may result from the homework, positive development during the session and other homework. The therapist highlighted how the newly acquired knowledge can be integrated into everyday life (or the therapist was very effective despite difficulties).

**8. Use of feedback and summaries**<br>**Key features:** The therapist should regularly ask for feedback to ensure his/her own understanding of the patient's situation as well as the patient's understanding of the therapy. The therapist should seek feedback in such a way that the patient does not feel tested and evaluated. The therapist can also summarize during the session.	
0	The therapist did not summarize and did not ask the patient for feedback to determine whether the patient has understood the matters discussed during the session.
1	*between 0 and 2*
2	The therapist made summaries but they were unclear and vague. The therapist encouraged the patient to give feedback but did not ask enough questions to ensure that the patient understood the reasoning or was satisfied with the session.
3	*between 2 and 4*
4	The therapist summarized during the session and asked enough questions in order to be sure that the patient understood the main points of the session. He adapted his behavior according to the feedback of the patient, where appropriate. Ideally, the session was summarized at the end. Minor problems were apparent (e.g., the therapist was contradictory or lecturing).
5	*between 4 and 6*
6	The therapist was especially good in identifying and responding to verbal and non-verbal feedback throughout the session (for example, he regularly checked the understanding of the patient). The main points of the session were summarized at the end, and the patient understood these summaries. Enough time was left for the therapist and patient to reflect on the session.

**9. Guided discovery**<br>**Key features: Through** the use of open-ended questions, the therapist helps the patient to explore his problems, to generate hypotheses regarding his current situation and to generate possible solutions on his own. In order to allow the patient to adopt a new way of looking at things, the therapist makes use of a Socratic question style, adapted to the developmental status of the adolescent as well as to the time point in therapy.	
0	No attempt was made for guided discovery. The therapist seemed to cross-examine the patient, whereby he forced the patient onto defensiveness or imposed his view on the patient.
1	*between 0 and 2*
2	There were minimal opportunities for guided discovery. Questions that target productive discovery were used only to a small extent.
3	*between 2 and 4*

4	By and large, the therapist helped the patient to open up new vistas through guided discovery (e.g., through the examination of evidence or the consideration of alternatives). The therapist asked his questions in an adequate manner. Minor problems were apparent (e.g., a contradictory, complicated or lecturing approach).
5	*between 4 and 6*
6	The therapist was especially skilled in asking open-ended questions in order to explore problems and to help the patient to draw his own conclusions. He reached a very good balance between skillful questioning and other forms of interventions. He was able to integrate interruptions or difficulties of the patient and to modify his course of action accordingly.

**10. Focus of cognitive model**<br>**Key features:** The therapist discusses cognitions (stuck points) and behaviors with the patient (particularly avoidance behaviors), which are relevant to the development and maintenance of the posttraumatic stress disorder. The therapist is able to assess or change the very same.	
0	The therapist did not assess specific thoughts, assumptions, and beliefs or the problematic behavior.
1	*between 0 and 2*
2	The therapist assessed the disorder-specific cognitions and the problematical behavior of the patient. The therapist had problems in setting a focus or he focused on cognitions and behaviors that are irrelevant for the central problem of the patient.
3	*between 2 and 4*
4	The therapist assessed and discussed specific negative beliefs and concrete sustaining processes of the patient. However, the therapist had trouble focusing on what is relevant or the focus was set on cognitions and behaviors that are irrelevant for the patient's core problem.
5	*between 4 and 6*
6	The therapist assessed and discussed central cognitions and behaviors that seem most promising for the patient's problems. The therapist, if necessary, made assessments of the assumptions and noted changes in beliefs.

**11. Rationale/transparency**<br>**Key features:** The therapist explains the structure of the therapy/session to the patient. The therapist follows and explains an explicit theoretical model that is age appropriate, so that the patient is able to understand it. For this he uses, for example, situations from the adolescent himself (e.g., when the patient becomes highly stressed, how he can recognize examples of the function of the trauma memory). The therapist provides an explanatory model that illustrates that it is important to reduce avoidance behavior, to admit memories and natural feelings and to verify the accuracy of beliefs of the patient in relation to the trauma and its consequences, as well as to change false beliefs. The therapist uses "mini-statements" that are relevant for the understanding of a particular topic or the implementation of a specific intervention during the session. Such explanations are introduced age appropriately (e.g., skills, emotions, dissociation, and maintenance of PTSD).	
0	The therapist used techniques without proper and explicit explanation. He did not attempt to give the patient an understanding of the techniques used.
1	*between 0 and 2*
2	The therapist tended to give either incomplete or unclear explanations of the techniques used.
3	*between 2 and 4*
4	The therapist gave clear and complete explanations and established an understanding with the patient.
5	*between 4 and 6*
6	The therapist gave very good explanations and provided a clear way to establish the patient's understanding.

**12. Selection of appropriate strategies**<br>**Key features:** The therapist tailors the choice of techniques and contents to the personality and disorder of the patient, the treatment goals, treatment phase and the course of the session. The therapist follows the treatment hierarchy (life-threatening behavior, therapy destroying behavior, etc.) and chooses an appropriate focus. In the section development tasks, the therapist is able to identify relevant subject areas together with the patient, as well as to set priorities. The focus is on the <u>quality of the choice</u> of therapeutic strategies, not on how effectively they are implemented. Note: Deviation from the prescribed treatment manual should only occur if necessary, but if required, can be regarded as competent, for example, if important current concerns regarding the treatment hierarchy have priority.	
0	The therapist did not choose an appropriate strategy (e.g., ignored the treatment hierarchy).
1	*between 0 and 2*

2	The therapist chose specific techniques, but the strategy as a whole was only vague or the techniques did not seem promising for this patient, or another content seemed to be more promising/necessary for this patient at this point of time.
3	*between 2 and 4*
4	The therapist followed a mainly coherent therapeutic strategy that seemed somewhat promising and included appropriate techniques for changing disorder-related cognitions and maintaining factors.
5	*between 4 and 6*
6	The therapist followed a coherent strategy that seemed very promising for this patient at this point of time and included the most appropriate techniques for key cognitions and the corresponding sustaining factors.

**13. Implementation of techniques**<br>**Key features:** The therapist implements the techniques in an effective manner, tailored to the disorder, personality and maturity of the patient, treatment goals, treatment phase and the course of the session. The focus is on how effectively the strategy was used. The therapist adjusts the interventions to the current state of development of the adolescent, considers the current mood and level of attention and is therefore flexible/adaptive in the implementation.	
0	The therapist implemented techniques inappropriately or in a wrong way.
1	*between 0 and 2*
2	The therapist used appropriate techniques but showed serious mistakes in their implementation.
3	*between 2 and 4*
4	The therapist used appropriate techniques and implemented them competently with few mistakes.
5	*between 4 and 6*
6	The therapist used appropriate techniques in a very competent and inventive way. The therapist made use of specific experimental techniques, which are aimed at core beliefs, in a very clever and inventive way, in the session as well as in the homework assignments. The therapist tailored methods successfully to the difficulties or challenges of the patient.

14. Homework setting<br>**Key features:** The therapist assigns homework that is adequate for the treatment phase, corresponding to the concept and pursuing clear goals.	
0	The therapist did not assign relevant homework.
1	*between 0 and 2*
2	The therapist assigned partially inadequate, vague or too general homework. The therapist assigned homework in an experienced manner without discussing it with the patient sufficiently and without revealing the logic behind the new homework.
3	*between 2 and 4*
4	The therapist discussed the homework with the patient appropriately with clear objectives and comprehensible logic. The homework not only was related to general topics but referred to specific cognitions of behaviors that were identified during the session. However, minor problems were apparent.
5	*between 4 and 6*
6	The therapist agreed on homework in a competent manner (patient understood the meaning and procedure; objective was clear), which allowed the patient to take new perspectives, to test hypotheses, to gain experience with new behaviors or to practice strategies (that were discussed during the session). The therapist anticipated difficulties with the patient in the implementation of homework, and solutions were determined.

Specific competencies for D-CPT

**15. Dealing with severe stress**<br>**Key features:** The therapist responds to the patient's current and reported situations of severe stress. Phase-typical suitable strategies are conveyed and applied. The therapist helps the patient to perceive, label and deal appropriately with tension and dysfunctional behaviors (e.g., dissociation, self-harm, addiction, suicidal tendency). If needed, the therapist introduces the use of stress reduction strategies at the beginning of the therapy. He/she identifies together with the patient triggers of severe stress and develops alternatives to the use of dysfunctional behaviors and techniques to regulate and tolerate stress. In the process, the therapist facilitates the independent application of learned techniques.<br>In the case that no severe stress occurs during the session or is not depicted, one can award a 4 or 5, as it can be assumed that the therapist was able to avoid severe stress through appropriate behaviors, i.e., previously adequately discussing appropriate coping strategies.	
0	The therapist ignored the patient's severe stress (in the session or reported).
1	*between 0 and 2*
2	The therapist responded to severe stress. There are however some difficulties apparent, e.g., the therapist was clearly too directive or was not directive enough in the beginning of the therapy or no appropriate strategies were acquired for dealing with severe stress.
3	*between 2 and 4*
4	The therapist responded to severe stress. Minor difficulties were apparent, for example, he was too directive in advanced treatment phases.
5	*between 4 and 6*
6	The therapist responded perfectly to the patient's severe stress. He/she enabled the patient to perceive severe stress and apply promising techniques.

**16. Dealing with emotions**<br>**Key features:** The therapist is able to respond appropriately to the patient's emotions: together with the patient, he/she identifies the emotion, labels it, i.e., lets the patient label it, and responds to it in an appropriate way. That means that in the case of appropriate (natural) feelings, e.g., anxiety, helplessness, sadness or anger, he/she validates and supports the patient in embracing and accepting it. In the case of avoidance of natural feelings, the therapist supports the patient to experience those (e.g., through detailed and interested questioning).<br>In the event of an inappropriate feeling, i.e., painful sequential feelings such as shame, guilt or (self-) hatred, he/she supports the patient to question that feeling. The therapist addresses the relationship, i.e., the difference, between thoughts and feelings (ABC-scheme).	
0	The therapist ignored central emotions (in session or reported by the patient).

1	*between 0 and 2*
2	The therapist assessed the emotions of the patient that did not appear to be significant to the patient's core problem or he/she did not deal appropriately with the central emotions.
3	*between 2 and 4*
4	The therapist assessed and addressed specific emotions of the patient but encountered difficulties, e.g., the therapist did not sufficiently support the patient in labeling/embracing the feeling.
5	*between 4 and 6*
6	The therapist assessed and addressed the central emotions and adequately supported the patient to address the emotions.

**17. Use of validation strategies**<br>**Key features:** Phase- and age-suitable validation strategies are applied. The dosage and use of the techniques are appropriate, and balance with changing-oriented interventions is maintained.<br>The therapist makes it clear that the patient's experience and behavior from his/her subjective view against the background of his life story makes sense (due to subjective experience or disorder because of activated assumptions), e.g., through attentive listening, exact reflection, paraphrasing, naming of emotions, verbalizing, radical authenticity.	
0	The therapist applied no phase- and age-suitable validation strategies. Or he/she did not succeed in creating balance between validation techniques and change-oriented interventions.
1	*between 0 and 2*
2	The therapist partly applied phase- and age-suitable validation strategies. He/she partially succeeded in creating balance between validation techniques and change-oriented interventions.
3	*between 2 and 4*
4	The therapist applied mainly phase- and age-suitable validation strategies. He/she succeeded most of the time in creating balance between validation techniques and change-oriented interventions.
5	*between 4 and 6*
6	The therapist applied optimal phase- and age-suitable validation strategies. He/she consistently succeeded in creating balance between validation techniques and change-oriented interventions.

**18. Use of change-oriented interventions**<br>**Key features:** Phase- and age-suitable change-oriented interventions are applied. The dosage and usage of techniques are appropriate, there is balance with validation strategies, and techniques for the facilitation of the acceptance of symptoms and situations are maintained. Change-oriented interventions can be cognitive (critical questioning, restructuring) and behavioral (application of skills in situations of severe stress).	
0	The therapist applied no phase- and age-suitable change-oriented interventions. Or he/she did not succeed in creating balance between change-oriented and validation techniques.
1	*between 0 and 2*
2	The therapist partly applied phase- and age-suitable change-oriented interventions. He/she partially succeeded in creating balance between change-oriented and validation techniques.
3	*between 2 and 4*
4	The therapist applied mainly phase- and age-suitable change-oriented interventions. He/she predominantly succeeded in creating balance between change-oriented and validation techniques.
5	*between 4 and 6*
6	The therapist applied optimal phase- and age-suitable interventions. He/she consistently succeeded in creating balance between change-oriented and validation techniques.

**19. Consideration of autonomy**<br>**Key features:** The therapist appropriately considers the adolescent's need for autonomy, i.e., facilitates autonomy in the case of reluctant adolescents. He/she shows no signs of trying too hard and leaves an appropriate amount of control.<br>Note: In psychoeducational sessions, less autonomy facilitating behaviors are also counted as competent.	
0	The therapist did not consider the adolescent's need for autonomy, e.g., he dominated the session or even left too much responsibility to the adolescent.
1	*between 0 and 2*
2	The therapist only partly considered the need for autonomy.
3	*between 2 and 4*
4	The therapist succeeded in considering the adolescent's need for autonomy; minor difficulties are apparent, e.g., on some occasions, the therapist takes control.

5	*between 4 and 6*
6	The therapist optimally succeeded in considering the adolescent's need for autonomy.

**20. Facilitating cooperation**<br>**Key features:** Therapist and patient both participate actively in the therapeutic process; the therapist must not dominate the session. A low extent of cooperation takes place when the therapist controls the session or, alternatively, leaves too much responsibility to the adolescent. To which extent is the definition of the problem/the solution carried out in cooperation with the adolescent? How strongly does the therapist motivate the adolescent to engage actively and explore their own questions, solutions and examples?<br>The therapist leaves the responsibility for participation to the patient.	
0	The therapist did not facilitate cooperation, e.g., he/she dominated the session or even left too much responsibility to the adolescent.
1	*between 0 and 2*
2	The therapist partly succeeded in facilitating cooperation.
3	*between 2 and 4*
4	The therapist succeeded in facilitating cooperation; minor difficulties were visible.
5	*between 4 and 6*
6	The therapist succeeded in optimally facilitating cooperation. The patient actively participated in the therapeutic process.

**21. Contingency management**<br>**Key features:** The therapist uses age-appropriate, authentic, immediate, behavior-related contingency management (verbally/ non-verbally) in order to reduce dysfunctional behavior, i.e., to constitute desirable behavior.<br>**-** minimally necessary attention, ignorance, time-out procedures, behavioral analyses, verbal expression, reduced body language (averted body language, breaking-up eye contact) for self-harming/suicidal behavior, dissociation, and self-devaluating comments<br>**-** maximally necessary social attention, complimenting, interested questioning, strengthened body language, e.g., reinforcing nodding, repetition and paraphrasing, telephone and mail contact to the therapist as an amplifier in cases of mastering difficult situations in assistance of skills, implementation of assignments, etc. In the case that there is no contingency management needed during the session and there is none apparent, assign a 4 or a 4 with regard to the severity of the patient.	
0	The therapist did not react to desirable/undesirable behavior according to the contingency management.
1	*between 0 and 2*

2	The therapist hardly reacted to desirable/undesirable behavior. The reactions were apparent from the view point of the observer but were not as explicit as they could be to influence the patient's behavior.
3	*between 2 and 4*
4	The therapist reacted in a reinforcing manner to desirable behavior and with extinction to undesirable behavior. Minor difficulties were apparent (e.g., the therapist could have reacted more explicitly or the therapist reacted too strongly and therefore provoked reactance of the patient).
5	*between 4 and 6*
6	The therapist optimally reacted according to the contingency management. He/she explicitly reinforced the patient's desirable behavior and reacted to undesirable behavior in a way that reduction is likely.
9	Not necessary in this session.

**22. Identification and modification of avoidance behavior**<br>The therapist names or works on avoidance behavior (e.g., emotional withdrawal) with respect to escape-strategies (dissociation, self-harm) of the patient during the session or off session (trauma-related avoidance of places/activities/people). If no avoidance behavior to be treated is noticeable, assign, depending on the severity of the patient, a 4 or a 5.	
0	The therapist ignored obvious avoidance behavior of the patient (in or off session).
1	*between 0 and 2*
2	The therapist barely responded to the avoidance behavior of the patient.
3	*between 2 and 4*
4	The therapist responded to the avoidance behavior of the patient. Minor difficulties were apparent (e.g., the therapist could specify the behavior more clearly as avoidance).
5	*between 4 and 6*
6	The therapist reacted in an optimal manner to the avoidance behavior of the patient. He named it or let the patient name it, integrated it into the disorder specific model of PTBS and helped the patient to actively counter it.

23. What is your overall impression of the therapist's competence in this session?
Key features:
The evaluation of this item touches upon the question of how competent the therapist uses the manual adapted to

- this specific patient (disorder, personality, intelligence),
- this therapy phase (degree of difficulties for interventions, directivity),
- this session (current problem, goals).

Is the therapist able to adapt his/her behavior to the specific difficulty, motivation as well as emotional instability of traumatized adolescent patients?
The statement about the therapist's competence relates to the current session, the concerned patient and the present manual. This does not constitute a "competence-judgment" in a global sense.

0......... ....1........ ....2....... ....3....... ....4....... …....5....... …......6

Poor	Average	Good	Very good

24. How engaged did the patient appear?
Key features: The patient's engagement during the therapy and while doing assignments is a central feature of the therapy's success. Its evaluation should be made independently of the evaluation of the patient's characteristics.
What matters is evaluating how engaged the patient is in the session, how elaborate he/she has done his/her assignments, how interested and involved he/she appeared and whether sufficient motivation for change exists.

0......... …1....... ....2....... ......3....... ....4......... …....5......... …......6

Very bad	Rather bad	Good	Very good

25. How difficult was the therapeutic work with this patient?
Key features: The patient's difficulty does not necessarily result from the degree of difficulty of the disorder; instead, it also constitutes his/her interpersonal style, understanding, openness towards psychological aspects, etc.
This item concerns the evaluation of how much the patient's characteristics influence the therapeutic work. Aggravating conditions on the patient's side could also include closeness, hostility, cognitive restrictions, and advanced avoidance as well as a high proportion of borderline-personality traits, multiple trauma-cluster and various factors that can make therapy especially difficult.

Point of origin for the evaluation should be 0. Depending on the conspicuousness the judgment is drawn towards 6.

0….. ……1…… ……2....... ……3…… ……4…… ……5…… ……6

Easy	Rather easy	Rather difficult	Extremely difficult

Gutermann, J., Schreiber, S., Matulis, S., Stangier, U., Rosner, R. & Steil, R. (2015). Therapeutic adherence and competence scales for Developmentally Adapted Cognitive Processing Therapy for adolescents with PTSD.
Citation: European Journal of Psychotraumatology 2015, 6: 26632 - http://dx.doi.org/10.3402/ejpt.v6.26632
