# Supplementary material for: Therapeutic adherence and competence scales for Developmentally Adapted Cognitive Processing Therapy for adolescents with PTSD
Source: Eur J Psychotraumatol. 2015 Mar 18;6:10.3402/ejpt.v6.26632. doi: 10.3402/ejpt.v6.26632 (PMC4366479; doi:10.3402/ejpt.v6.26632)
Supplement: Therapeutic adherence and competence scales for Developmentally Adapted Cognitive Processing Therapy for adolescents with PTSD [file EJPT-6-26632-s004.pdf]

Information on Developmentally Adapted Cognitive Processing Therapy (D-CPT)

In phase I, the patient and therapist agree on the goals and rules of therapy and develop an emergency plan. In phase II, the patient learns and acquires strategies for handling intense emotion and distress. In phase III, the patient is encouraged to challenge over-generalizing thoughts derived from the traumatic experience, which are called “stuck points”. In the last phase, the patient and therapist address topics that are important for the adolescent’s development, such as finding a non-offending partner or planning education and vocational training.

For a detailed description, see:

Matulis, S., Resick, P. A., Rosner, R., & Steil, R. (2014). Developmentally adapted cognitive processing therapy for adolescents suffering from posttraumatic stress disorder after childhood sexual or physical abuse: A pilot study. *Clinical Child and Family Psychology* Review, 17(2), 173-190.
