# Supplementary material for: Therapeutic adherence and competence scales for Developmentally Adapted Cognitive Processing Therapy for adolescents with PTSD
Source: Eur J Psychotraumatol. 2015 Mar 18;6:10.3402/ejpt.v6.26632. doi: 10.3402/ejpt.v6.26632 (PMC4366479; doi:10.3402/ejpt.v6.26632)
Supplement: Therapeutic adherence and competence scales for Developmentally Adapted Cognitive Processing Therapy for adolescents with PTSD [file EJPT-6-26632-s005.pdf]

Inclusion and exclusion criteria of the pilot trial of Developmentally Adapted Cognitive Processing Therapy (D-CPT)

Inclusion criteria of the pilot trial of D-CPT included being between 13 and 21 years of age, PTSD as a primary diagnosis after CSA/CPA, safe living conditions and sufficient knowledge of German. Informed consent was received from both the parents/legal guardians (if under 18) and the participant. Exclusion criteria were acute suicidality or life-threatening self-harming behavior within the last six months, substance-related or organic mental disorder, pervasive developmental disorder, acute or lifetime diagnosis of a psychotic disorder, lifetime diagnosis of bipolar disorder, current diagnosis of substance dependence (abstinence < six months), mental retardation (IQ ≤ 75), simultaneous psychological or psychiatric treatment and variable psychopharmacological medication use.
